# Influenza Vaccination of Healthcare Workers: Critical Analysis of the Evidence for Patient Benefit Underpinning Policies of Enforcement

**DOI:** 10.1371/journal.pone.0163586

**Published:** 2017-01-27

**Authors:** Gaston De Serres, Danuta M. Skowronski, Brian J. Ward, Michael Gardam, Camille Lemieux, Annalee Yassi, David M. Patrick, Mel Krajden, Mark Loeb, Peter Collignon, Fabrice Carrat

**Affiliations:** 1 Institut national de santé publique du Québec, Quebec City, Quebec, Canada; 2 Laval University, Quebec City, Quebec, Canada; 3 British Columbia Centre for Disease Control, Vancouver, British Columbia, Canada; 4 University of British Columbia, Vancouver, British Columbia, Canada; 5 Research Institute of the McGill University Health Centre, Montreal, Quebec, Canada; 6 University of Toronto, University Health Network, Toronto, Ontario, Canada; 7 McMaster University, Faculty of Health Sciences, Hamilton, Ontario, Canada; 8 Australian National University, Acton, Australia; 9 Canberra Hospital, Garran, ACT, Australia; 10 Institut National de la Santé et de la Recherche Médicale, Institut Pierre Louis d’Epidémiologie et de Santé Publique, Paris, France; 11 Sorbonne Universités, Institut Pierre Louis d’Epidémiologie et de Santé Publique, Paris, France; 12 Assistance Publique-Hôpitaux de Paris, Hôpital Saint Antoine, Unité de Santé Publique, Paris, France; University of Chieti, ITALY

## Abstract

**Background:**

Four cluster randomized controlled trials (cRCTs) conducted in long-term care facilities (LTCFs) have reported reductions in patient risk through increased healthcare worker (HCW) influenza vaccination. This evidence has led to expansive policies of enforcement that include all staff of acute care hospitals and other healthcare settings beyond LTCFs. We critique and quantify the cRCT evidence for indirect patient benefit underpinning policies of mandatory HCW influenza vaccination.

**Methods:**

Plausibility of the four cRCT findings attributing indirect patient benefits to HCW influenza vaccination was assessed by comparing percentage reductions in patient risk reported by the cRCTs to predicted values. Plausibly predicted values were derived according to the basic mathematical principle of dilution, taking into account HCW influenza vaccine coverage and the specificity of patient outcomes for influenza. Accordingly, predicted values were calculated as a function of relevant compound probabilities including vaccine efficacy (ranging 40–60% in HCWs and favourably assuming the same indirect protection conferred through them to patients) × change in proportionate HCW influenza vaccine coverage (as reported by each cRCT) × percentage of a given patient outcome (e.g. influenza-like illness (ILI) or all-cause mortality) plausibly due to influenza virus. The number needed to vaccinate (NNV) for HCWs to indirectly prevent patient death was recalibrated based on real patient data of hospital-acquired influenza, with adjustment for potential under-detection (5.2-fold), and using favourable assumptions of HCW-attributable risk (ranging 60–80%).

**Results:**

In attributing patient benefit to increased HCW influenza vaccine coverage, each cRCT was found to violate the basic mathematical principle of dilution by reporting greater percentage reductions with less influenza-specific patient outcomes (i.e., all-cause mortality > ILI > laboratory-confirmed influenza) and/or patient mortality reductions exceeding even favourably-derived predicted values by at least 6- to 15-fold. If extrapolated to all LTCF and hospital staff in the United States, the prior cRCT-claimed NNV of 8 would implausibly mean >200,000 and >675,000 patient deaths, respectively, could be prevented annually by HCW influenza vaccination, inconceivably exceeding total US population mortality estimates due to seasonal influenza each year, or during the 1918 pandemic, respectively. More realistic recalibration based on actual patient data instead shows that at least 6000 to 32,000 hospital workers would need to be vaccinated before a single patient death could potentially be averted.

**Conclusions:**

The four cRCTs underpinning policies of enforced HCW influenza vaccination attribute implausibly large reductions in patient risk to HCW vaccination, casting serious doubts on their validity. The impression that unvaccinated HCWs place their patients at great influenza peril is exaggerated. Instead, the HCW-attributable risk and vaccine-preventable fraction both remain unknown and the NNV to achieve patient benefit still requires better understanding. Although current scientific data are inadequate to support the ethical implementation of enforced HCW influenza vaccination, they do not refute approaches to support voluntary vaccination or other more broadly protective practices, such as staying home or masking when acutely ill.

## Introduction

Annual influenza vaccination for health care workers (HCWs) is widely endorsed [1–5] and increasingly enforced on the basis that it will reduce influenza-associated morbidity and mortality in patients [6–10]. Four cluster randomized controlled trials (cRCTs), conducted exclusively in long-term care facilities (LTCFs) and nursing homes have specifically assessed indirect patient benefits from HCW influenza vaccination and have been most often cited in the support of mandatory HCW policies [11–14]. Two pivotal systematic reviews and meta-analyses have been published summarizing and pooling these four cRCT findings, but reached different conclusions about the strength of that evidence [15–18]. Whereas the review conducted by investigators of the United States Centers for Disease Control and Prevention (US CDC) [15] based on the GRADE framework characterized the overall quality of evidence as moderate [19], the Cochrane review concluded that the evidence was insufficient to support HCW influenza vaccination as an approach to reduce patient risk [16–18]. Such uncertainty in the quality of the evidence warrants closer examination. This is particularly important given that compulsory or coercive (e.g. vaccinate-or-mask) policies have been extrapolated in some jurisdictions to not only include HCWs providing direct patient care in LTCFs, but also to include all staff in acute-care hospitals and other healthcare settings [6–10].

The ethical premise for mandatory HCW influenza vaccination critically hinges upon the valid demonstration of patient benefit substantial enough to justify infringement of the personal rights of HCWs who would otherwise choose not to receive influenza vaccine each year. In this re-analysis, we undertake critique and quantification of the evidence for indirect patient benefit, translating methodological considerations in a way not previously made plain or accessible. In particular, our critique evaluates the plausibility of cRCT conclusions by assessing consistency with the natural boundaries for indirect vaccine effects set by the mathematical principle of dilution, taking into account involved compound probabilities. In addition, to gauge the potential impact of enforced HCW vaccination if further extended to all acute care hospitals, we quantify the number needed to vaccinate (NNV) to potentially prevent a single patient death in the hospital setting—a core epidemiological concept that is critical to policy-makers but that has not previously been addressed in the scientific literature.

## Methods

### a. Core concepts: vaccine efficacy (VE), outcome specificity, and the principle of dilution

Vaccine efficacy (VE) is the percentage reduction in a particular disease outcome in vaccinated compared to unvaccinated individuals. For example, a VE of 60% means that vaccinated people have a 60% reduction in their risk of a given outcome compared to unvaccinated people.

Studies of VE may include a variety of outcomes for which prevention may be desirable. For example, for influenza, preventable outcomes of interest may include laboratory-confirmed influenza infection, or clinical influenza-like illness (ILI), or hospitalization or death. Illness that is laboratory-confirmed for influenza using molecular diagnostic tools (such as polymerase chain reaction (PCR)) is the most specific study outcome and virtually 100% of cases defined this way are actually related to the influenza virus (i.e. the positive predictive value for influenza in PCR-positive cases is nearly 100%). But it may be challenging to study laboratory-confirmed outcomes and so other surrogate clinical outcomes are sometimes used in VE studies. For example, ILI is typically defined as a clinical syndrome consisting of some constellation of respiratory and systemic symptoms, such as cough and fever, but these are not just features of influenza. ILI can be caused by multiple other non-influenza respiratory pathogens against which influenza vaccine has no effect. When assessing the benefits of influenza vaccine, these other causes of ILI dilute the measured effects. The proportion of seasonal ILI cases actually due to influenza (i.e. positive predictive value for influenza) may vary with defining conditions but is generally <35% [20]. As a more extreme example, the tally of all deaths without laboratory confirmation and regardless of cause (i.e. “all-cause mortality”) includes a vast array of non-influenza etiologies, including not only other infections, but also a multitude of other causes such as cancer, accidents, trauma, cardiovascular events, metabolic conditions, kidney disease etc. The positive predictive value for influenza is even smaller (i.e. more diluted) when using “all-cause mortality” than when using ILI as a study outcome.

It may be of public health interest to assess the benefits of vaccine against the sum total of any ILI or mortality, but to be considered valid these claimed benefits must adhere to the basic principle of dilution. The principle of dilution governs the inverse relationship between percentage reduction in a given outcome, and percentage contribution of non-targeted events to that outcome. Because of dilution effects, the more that non-target conditions contribute to an outcome the lower must be the percentage reduction in that outcome brought about by a targeted intervention. This basic mathematical principle of dilution is universal; it applies not only to vaccination but also to other practical considerations involving percentage reductions. For instance, most shoppers intuitively know that the percentage reduction from a discount coupon will always be greatest in relation to the price of the product featured on the coupon, and lower when calculated in relation to their entire shopping bill that includes other non-applicable items. This commonplace example is shown in the Supporting Information to reinforce the general truism of the basic principle of dilution (S1 Appendix).

Influenza vaccination targets influenza virus and is ineffective against non-influenza causes of ILI, hospitalization or death. The mathematical principle of dilution thus requires that the percentage reduction (i.e. VE) must be lower when non-targeted events (i.e. non-influenza etiologies) are included in the study outcome (e.g. ILI or all-cause mortality) than when only the targeted condition (i.e. influenza infection) contributes. In other words, the lower the positive predictive value of an outcome for influenza, the lower must be the measured VE for that outcome. Under valid conditions, VE will always be higher for laboratory-confirmed influenza than for ILI, and higher for ILI than for all-cause mortality. Where this pattern is not observed, claimed benefits of a vaccine cannot be accepted at face value; other explanatory factors must exist.

In fact, the percentage reduction in a given outcome can never exceed the positive predictive value for influenza of that outcome (even with an influenza VE of 100%), and will be even lower when further taking into account vaccine coverage. When comparing two groups or populations in order to attribute vaccine benefits, the percentage reduction in a given outcome will be proportional to the absolute difference (Δ) in vaccine coverage between the two populations compared.

The percentage reduction in a given outcome attributed to change in vaccination coverage can thus be calculated according to the following equation, based upon the principle of dilution:

%reductioninoutcome=(%vaccineefficacy)x(%influenzainoutcome)x(Δ%vaccinecoverage) (1)

### b. Critique of cRCT evidence for patient benefit

Through Medline search, we confirmed that the US CDC (published 2014 [15]) and Cochrane (published 2010 [16], 2013 [17] and 2016 [18]) reviews captured all RCTs that specifically assessed indirect patient benefit through HCW influenza vaccination. In the four identified cRCTs, multiple patient outcomes were assessed, ranging from most-to-least specific for influenza (e.g. laboratory-confirmed influenza versus ILI versus all-cause mortality). The 2010 [16] and not later versions of the Cochrane review is used here because for the all-cause mortality outcome the 2013 version [17] excluded the Hayward trial [13] and the 2016 version [18] did not present pooled analysis.

Using the principle of dilution to anchor our analyses and interpretations, we assessed the plausibility of the cRCT results in attributing patient disease reductions to HCW influenza vaccination. Where the pattern of highest-to-lowest percentage reduction against most-to-least specific outcomes (i.e. laboratory-confirmed influenza > ILI > all-cause mortality) was not observed, or the percentage reduction was not proportional to the change in vaccine coverage, a *de facto* flaw in the exclusive attribution of vaccine effects was understood. To then quantify the potential magnitude of discrepancy in attributing patient outcome reductions to HCW vaccination, we derived the ratio of cRCT-reported versus derived-predicted reductions.

Predicted reductions in patient outcomes were derived according to *Eq 1*. For this analysis, we assumed a VE of 60% as reported in an earlier meta-analysis of RCTs in young adults that used virologically-confirmed influenza outcomes [21]; however, we also explored for VE of 40%. We conservatively assumed that the direct VE in HCWs would provide equivalent indirect protection to patients. We assumed that across the influenza season the percentage of true influenza was 100% in laboratory-confirmed influenza, 35% in ILI (double that reported by Carman and comparable to or higher than other published estimates) [12,20,22] and 10% in all-cause mortality [23]. Finally, we used the absolute difference (Δ) in HCW influenza vaccine coverage between control and intervention sites as reported by each of the cRCTs.

### c. Quantification of the HCW NNV to indirectly prevent patient death

NNV is usually defined as (1/(absolute risk reduction)) where the absolute risk reduction is the difference in incidence between unvaccinated and vaccinated individuals.

For the indirect prevention of patient death through HCW influenza vaccination, the absolute risk reduction is the number of patient deaths that are attributable to hospital-acquired influenza and that are also preventable by HCW vaccination, divided by the number of unvaccinated HCWs who may have caused these deaths. The NNV for HCW influenza vaccination to prevent patient death due to hospital-acquired influenza is thus derived as:

$$NNV=\frac{1}{Number\;of\;patient\;deaths\;preventable\;by\;HCW\;vaccination/Number\;of\;unvaccinated\;HCWs}$$

or:

$$NNV=\frac{Number\;of\;unvaccinated\;HCWs}{Number\;of\;patient\;deaths\;preventable\;by\;HCW\;vaccination}$$ (2)

To parameterize the NNV estimate for acute care, we used two large recent studies in the US (Jhung et al) [24] and Canada (Taylor et al) [25]. We assumed that 60% of all hospital-acquired influenza was attributable to HCWs, recognizing patients and visitors are other contributing sources [26–28], and again allowed direct VE of 60% in HCWs [21] and the same indirect protection to be conferred through them to patients.

## Results

### a. Critique of cRCT evidence for patient benefit in LTCFs

#### i. Evaluation in the context of the principle of dilution

**Fig 1:**
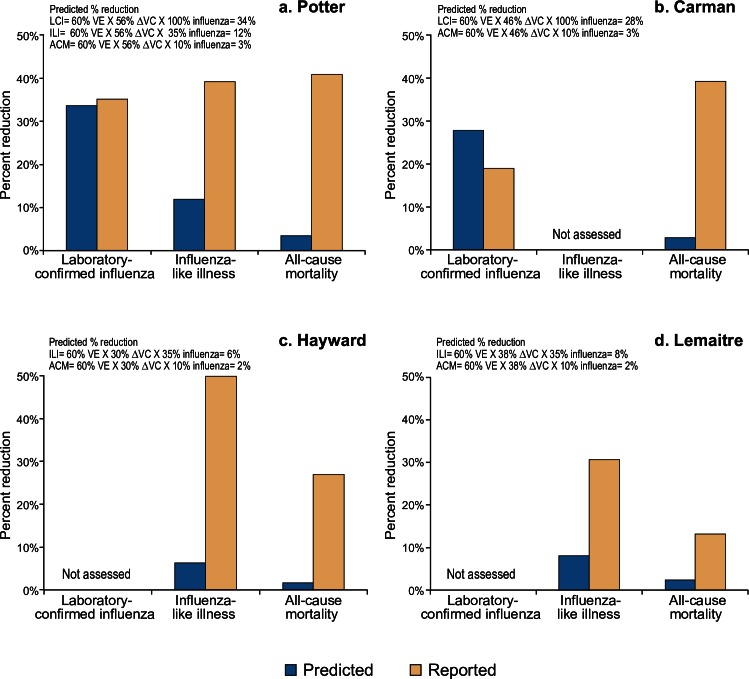
Reported and predicted percentage reductions in patient outcomes between intervention and control sites among cluster randomized controlled trials to assess indirect patient benefits from increased influenza vaccine coverage of healthcare workers in long-term care facilities. Abbreviations: VE = vaccine efficacy; Δ VC = absolute difference in vaccine coverage; LCI = laboratory-confirmed influenza; ILI = influenza-like illness; ACM = all-cause mortality

As shown in Table 1 and Fig 1, Potter [11] and Carman [12] reported greater percentage reductions with less specific patient outcomes (i.e. percentage reduction in all-cause mortality > ILI > laboratory-confirmed influenza), a reversal of the trend required for plausibility according to the principle of dilution. For laboratory-confirmed influenza, the reported percentage reduction was comparable to or lower than predicted (Potter 35% vs 34%, respectively; Carman 19% vs 28%, respectively) (Table 1, Fig 1). In contrast, the reported percentage reductions for non-specific outcomes were several-fold higher than predicted, exceeding values that could be reasonably attributed to vaccine effects in all four cRCTs (Table 1, Fig 1). Compared to predicted values, reported reductions in patient ILI outcomes were 8-fold higher in the Hayward trial during the 2003–04 season [13], 4-fold higher in the Lemaitre trial [14] and 3-fold higher in the trial by Potter [11] (Table 1, Fig 1). For all-cause mortality there was even greater discrepancy; reported percentage reductions attributed to HCW influenza vaccination were 12-15-fold greater-than-predicted for Potter, Carman, and Hayward, and 6-fold greater in the trial by Lemaitre.

**Table 1:** Reported and predicted percentage reductions in patient outcomes between intervention and control sites among cluster randomized controlled trials to assess indirect patient benefits from increased influenza vaccine coverage of healthcare workers in long-term care facilities.

	HCW Influenza Vaccine Coverage and Absolute Difference (Δ%)	Patient Outcomes		
Publication		Laboratory-confirmed influenza	Influenza-like Illness (ILI)	All-cause mortality
**cRCTs—Author [Reference] Publication Year, Country (Number of Residents, Sites; Analysis Period; Duration)**				
**Potter [11] 1997 Scotland (1059 residents, 12 sites; end October 1994—end March 1995; 5 months)**				
Incidence intervention vs. control	61% vs. <5%	4.2% vs. 6.5%	4.5% vs. 7.4%	10.2% vs. 17.2%
Reported ΔVC^a^ and relative % reduction^b^	Δ 56%	35.2%	39.2%	40.8%
Predicted relative % reduction^c^	NA	33.6%	11.8%	3.4%
Ratio of reported / predicted relative % reduction	NA	1.0	3.3	12.1
**Carman [12] 2000 Scotland (1427 residents, 20 sites; November 18, 1996—March 31, 1997; 4·5 months)**				
Incidence intervention vs. control	51% vs. 5%	5.4% vs. 6.7%^d^	NR	13.6% vs. 22.4%
Reported ΔVC^a^ and relative % reduction^b^	Δ 46%	18.9%	NR	39.2%
Predicted relative % reduction^c^	NA	27.6%	NR	2.8%
Ratio of reported / predicted relative % reduction	NA	0.7	NR	14.2
**Hayward [13] 2006 United Kingdom, excluding Scotland and Wales**				
**Year 1 (2604 residents, 44 sites; November 3, 2003—~January 18/25, 2004; ~2·5 months)**^e^				
Incidence intervention vs. control	35% vs. 5%	NR	11.4% vs. 22.7%	11.2% vs. 15.3%
Reported ΔVC^a^ and relative % reduction^b^	Δ 30%	NR	49.9%	26.9%
Predicted relative % reduction^c^	NA	NR	6.3%	1.8%
Ratio of reported / predicted relative % reduction	NA	NR	7.9	15.0
**Year 2 (2661 residents, 44 sites; ~January 2, 2004—~February 20/27, 2005; ~1·5 months)**^e^				
Incidence intervention vs. control	31% vs. 4%	NR	12.1% vs. 13.3%	8.0% vs. 9.1%
Reported ΔVC^a^ and relative % reduction^b^	Δ 27%	NR	8.8%	11.9%
Predicted relative % reduction^c^	NA	NR	5.7%	1.6%
Ratio of reported / predicted relative % reduction	NA	NR	1.6	7.3
**Lemaitre [14] 2009 France (3400 residents, 40 sites; January 1, 2007—March 18, 2007; 2·5 months)**				
Incidence intervention vs. control	70% vs. 32%	NR	6.7% vs 9.7%	5.2% vs 6.0%
Reported ΔVC^a^ and relative % reduction^b^	Δ 38%	NR	30.7%	13.3%
Predicted relative % reduction^c^	NA	NR	8.0%	2.3%
Ratio of reported / predicted relative % reduction	NA	NR	3.8	5.8
**Summary pooled estimates from meta-analyses**				
**United States Centers for Disease Control and Prevention [15]**				
Incidence intervention vs. control sites	NA	5.1% vs. 6.4%	8.1% vs. 14.1%	9.0% vs. 13.0%
Reported relative % reduction (95% CI)^f^	NA	20% (-108%, 69%)	42% (27%, 54%)	29% (15%, 41%)
**Cochrane Collaboration [16]**				
Incidence intervention vs control sites	NA	4.5% vs. 5.3%	8.1% vs. 11.4%	9.0% vs. 13.0%
Reported relative % reduction (95% CI)^f^	NA	14% (-68%, 56%)	29% (10%, 45%)	34% (21%, 45%)

HCW = Healthcare worker; VC = vaccine coverage; cRCT = cluster randomized controlled trial; NA = Not applicable; NR = Not reported; CI = confidence interval.

a. Percentage difference (increase) displayed for influenza vaccine coverage in HCWs is absolute difference (Δ) between intervention and control sites.

b. Reduction displayed for outcomes of laboratory-confirmed influenza, ILI and all-cause mortality is the relative percentage reduction in intervention vs. control sites derived as: $\frac{\left(incidence\;control\;sites-incidence\;intervention\;sites\right)}{incidence\;control\;sites}\;x\;100\%$.

c. Predicted relative % reduction was calculated as: (*Vaccine efficacy*) *x* (% *influenza in outcome*) *x* Δ *vaccine coverage* assuming that vaccine efficacy is 60% and % influenza in the outcome is 100% for laboratory-confirmed influenza, 35% for ILI and 10% for all-cause mortality, across the full surveillance periods shown.

d. Among a subset of 39 patients in “no vaccine” hospitals with symptoms consistent with influenza or upper respiratory tract infection (per Carman et al’s unspecified ILI definition), 6/39 (15%) were reverse-transcriptase PCR positive for influenza.

e. Influenza period used for analysis not specified but indicated to span from the date that ILI reporting through the Royal College of General Practitioners’ sentinel surveillance exceeded 30/100,000 and until one week after this rate returned below that threshold for ILI, two weeks for all-cause mortality. Dates shown are approximated from Hayward et al.’s Fig 1 [13].

f. Reduction displayed for outcomes of laboratory-confirmed influenza, ILI and all-cause mortality is the relative percentage reduction in intervention vs. control sites, derived from the reported risk ratios as: (1 − *risk ratio*) *x* 100%.

Reported versus predicted values for percentage reduction in laboratory-confirmed influenza, influenza-like illness and all-cause mortality among long-term care facility residents are displayed for the four cluster randomized controlled trials to assess indirect benefits of increased healthcare worker influenza vaccination, including: (a) Potter et al [11]; (b) Carman et al [12]; (c) Hayward et al [13]; and (d) Lemaitre et al [14]. Assumptions and derivations for predicted values accompany each study panel.

#### ii. Period of analysis exposes cRCT bias and error

In two [11,14] of the four cRCTs, the period of analysis preceding influenza onset exposes the presence of a bias that may contribute to the difference between reported and predicted reductions.

Based on community surveillance, Potter reported outbreaks of influenza A and B beginning in calendar week 6 and week 1, respectively, of 1995, which were 100 and 60 days, respectively, into the follow-up period that started November 1^st^ 1994 [11]. More than half of the difference in the cumulative mortality curves between intervention and control sites had already accrued before the onset of influenza activity from day 60—a period before which non-influenza etiologies, not preventable by influenza vaccination, would have been the more likely cause of death. The non-vaccine factors reducing mortality before the onset of influenza likely continued to play a role beyond day 60 to the end of the follow-up period, further compounding the misattribution of mortality reduction to HCW influenza vaccination even during the influenza period.

Lemaitre et al also acknowledged that rates were lower in their intervention group compared to the control group preceding the influenza peak by 9 weeks for all-cause mortality and 4 weeks for ILI, a between-group difference they suggested was likely due to an early peak in circulation of RSV, against which influenza vaccine has no effect [14]. During the more relevant period of actual influenza circulation, rates of all-cause mortality and ILI were similar between the study groups, with no significant reductions based on HCW vaccination and no difference at the peak of influenza transmission.

While there may have been *some* sporadic influenza activity before the recognized influenza period, it is unlikely that *most* of the sustained influenza risk in these studies could have otherwise preceded (or gone completely undetected by) the community surveillance systems. In that context, most of the all-cause mortality difference reported by these studies before the influenza period would have been a measure of bias or confounding, rather than of influenza vaccine effects.

#### iii. Hayward cRCT [13]

The cRCT by Hayward et al was commissioned as a more definitive test of the earlier trial findings and as such warrants closer scrutiny [13]. This cRCT was conducted over two seasons (2003–4 and 2004–5) during which comparable increments in HCW vaccine coverage between intervention and non-intervention sites (2003–04: Δ = 30% and 2004–05: Δ = 27%) were achieved. Reductions in ILI and all-cause mortality were significant during the 2003–04 season but substantially lower and non-significant the following season (Table 1). The authors mostly dismissed the second-season findings (deriving an NNV based on first season only) on the rationale that influenza activity had been exceptionally low in 2004–05. The CDC and Cochrane authors also excluded the second season from the pooled estimate of their meta-analyses for unclear reasons [15,16]. Since the second season of Hayward has not been influential on policy decisions we have also not considered it in the current analysis, although it is worth noting that surveillance reports show the 2004–05 season to have been comparable to and more representative of most other prior seasons since 2000 [29–31].

Hayward et al stratified their analyses based on influenza and no-influenza periods with significant benefits during the 2003–04 season reported only during the influenza period. The 30% absolute difference (Δ) in vaccine coverage in HCWs may have potentially improved protection against laboratory-confirmed influenza infection in otherwise susceptible HCWs by up to just 18% (i.e. *Eq 1*: 60% X 100% X 30%). Similarly based on *Eq 1*, we may have expected corresponding reductions of up to 6% in ILI and 2% in all-cause mortality given proportionate contributions of influenza to these less specific outcomes (35% and 10%, respectively) and their dilution otherwise by non-vaccine-preventable etiologies (Table 1, Fig 1). Instead, the authors reported 50% reduction in ILI and 27% reduction in all-cause mortality (Table 1, Fig 1). Even on the basis of predicted reductions that are optimistic, approximately 90% of the reductions in patient disease reported by the Hayward cRCT cannot be plausibly explained by HCW vaccination, as shown below:

$$\;Proportion\;ILI\;unexplained=\frac{50\%\;reported-6\%\;predicted}{50\%\;reported}=88\%$$ (3)

$$\;Proportion\;all\;cause\;mortality\;unexplained=\frac{27\%\;reported-2\%\;predicted}{27\%\;reported}=93\%$$ (4)

Even under the further extreme assumption that as much as 50% of ILI and 30% of all-cause mortality might have been due to influenza across the ~2.5 month follow-up period, the HCW vaccine intervention could have only reduced these outcomes by 9% and 5%, respectively (five times lower than reported by Hayward et al), leaving 80% or more of the reported reductions still unexplained by HCW vaccination. Such large reductions in patient risk attributed to HCW influenza vaccination are even more unbelievable when considering that the 2003–04 season was a notorious vaccine mismatch season during which the A(H3N2) variant A/Fujian/411/2002 dominated and was antigenically distinct from the A/Moscow/10/1999-like vaccine strain for which VE <60% was then considerably more likely [32].

The ILI case definition used by Hayward et al was adjusted to be more sensitive in identifying as many cases as possible among elderly patients [13]. The authors allowed any respiratory symptom or sign in the context of a new or worsening condition of any kind to be counted as ILI. Ultimately, there were 300 ILI cases in control homes and 142 in intervention homes. There were 19 and 13 deaths, respectively associated with their broad ILI definition, providing respective ILI case fatalities of 6% (19/300) and 9% (13/142). The total number of residents (denominator) was similar in the intervention and control homes, allowing focus on numerator tallies alone. As shown in **Table 2** adapted from the Hayward trial, the number of all-cause deaths in the control homes was 203 and in the intervention homes was 140, an excess of 63 all-cause deaths in the control homes of which just 6 (i.e. 19–13) were associated with ILI.

**Table 2:** Mortality tallies during the defined influenza analysis period among residents of care homes with and without an intervention to increase healthcare worker influenza vaccination, reproduced from the 2003–04 season of the Hayward cluster randomized controlled trial [13].

	Control homes	Intervention homes	
	N = 1323 residents	N = 1249 residents	Difference
	Number of deaths	Number of deaths	
All-cause mortality	203	140	63
Death with ILI^*^	19	13	6
Death with no ILI*	184	127	57

* ILI = influenza-like illness, defined as fever of ≥37.8C (oral), OR an acute deterioration in physical or mental ability, plus either new onset of one or more of respiratory symptoms OR an acute worsening of a chronic condition involving respiratory symptoms.

If all of the excess all-cause mortality in the control homes was due to influenza as assumed by the authors, then it would also require that 90% (i.e. 57/63) of the influenza deaths occurred in the absence of ILI—i.e. without any respiratory symptom or sign as defined by the enhanced surveillance protocol for this study. Unless there was profoundly poor compliance with the study protocol in reporting secondary outcomes of ILI or ILI with death, there must have instead been a serious misattribution of patient mortality to influenza to explain these findings. Although ILI and mortality surveillance were based on weekly aggregate reporting and could not be individually-linked, the aggregate ILI fatalities in the Hayward study were similar to other findings [25] and as such, under-reporting of ILI (and notably in relation to deaths) does not appear to have been a particular issue. While influenza in older people can occur without fever, to accept that nearly all (i.e. 90%) influenza infections in the elderly lack any respiratory feature, despite almost exclusive replication in the respiratory epithelium, would otherwise require a paradigm shift in the current understanding of this disease. Either explanation for these paradoxical findings reinforces concerns about the reliability and validity of the study’s conclusions, particularly in relation to policies to coerce or compel HCW influenza vaccination.

### b. HCW NNV to indirectly prevent patient death

In their conclusions, Hayward et al summarized that just eight HCWs would need to be vaccinated to prevent one patient death (i.e. number-needed-to-vaccinate [NNV] = 8) and further proposed in their concluding statements that their results were likely to be generalizable to other care homes and acute hospital settings, in particular geriatric and rehabilitation wards [13].

To put this summary value and conclusion into context, if actually generalized to the ~1.7 million HCWs in LTCFs in the US, Hayward’s estimate of the NNV would mean that 212,500 patient deaths in LTCFs could be prevented every year through HCW vaccination (i.e. 1.7 million/8), an estimate that substantially exceeds the total number of influenza deaths estimated annually for the whole US population (3000–49,000) [33,34]. If further extrapolated to the ~5.5 million hospital workers in the US it would mean that an additional 687,500 patient deaths could be prevented each year—more even than the 675,000 influenza deaths estimated to have occurred in the whole of the US during the 1918 Spanish influenza pandemic [35,36]. Some attempt at recalibration of this NNV is clearly warranted but, surprisingly, has not previously been proposed in the scientific literature.

To re-assess the NNV for acute care we therefore drew on two networks that have published estimates of hospital-acquired influenza—one in the US and one in Canada. The US FluSurv-Net reported by Jhung et al was conducted during the 2010–11 season and covers a population under surveillance of 29 million (~10% of the US population) [24]. We estimated the number of associated HCWs to be 10% of the 5.5 million hospital workers in the US (i.e. 550,000) and assumed 70% vaccine coverage, as documented elsewhere for US hospitals [37]. The study identified 6,171 laboratory-confirmed-influenza cases of which 172 (2.8%) were hospital-acquired and 27 (16%) died (Table 3).

**Table 3:** Number of hospital-acquired influenza (HAI) cases and deaths due to unvaccinated health care workers (HCWs), and number (of HCWs) needed to vaccinate (NNV) per patient HAI death averted during the 2010–11 season based on hospital networks in the United States and Canada.

	Jhung [*23*]	Taylor [*24*]
	United States	Canada
Number of HCWs	550,000^a^	113,000^b^
Vaccine coverage (VC) in HCWs	70%^c^	40%^d^
**Number of unvaccinated HCWs**^**e**^	**165,500**	**67,800**
Patient HAI cases reported^f^	172^g^	82^h^
Patient HAI deaths reported	27^g^	7.3^i^
Patient HAI deaths attributed to any HCW^j^	16.2	4.4
Patient HAI deaths attributed to unvaccinated HCW^k^	8.4	3.5
**Patient HAI deaths preventable by HCW vaccination**^**l**^	**5.0**	**2.1**
Number of HCW needed to vaccinate (NNV) per patient HAI-death prevented^m^	32,819	32,688

HCW = healthcare worker; HAI = hospital-acquired influenza; NNV = number needed to vaccinate.

**Bolded values** represent the numerator and denominator directly used in the NNV calculation (per footnote (m) below).

a. Estimated as 10% of the total number of hospital workers for the US (i.e. 10% × 5,500,000 = 550,000).

b. Estimated as 35% of the total number of hospital workers for Canada (i.e. 35% × 500,000 = 175,000) included in the Taylor network in total, and 65% (i.e. 35/54) of the hospitals in the network contributing during the 2010–11 season (i.e. 65% X 175,000 = 113,000).

c. Based on documented vaccine coverage among HCWs in US hospitals [37].

d. Based on documented vaccine coverage among HCWs in Canadian hospitals [38,39].

e. Derived as: *number of HCWs x* (100% − *VC*); where VC = vaccine coverage.

f. HAI defined by Taylor et al as symptom onset 96 hours or longer after admission or readmission with a positive test result less than 96 hours after discharge or a positive test result less than 96 hours after transfer from another facility and by Jhung et al as laboratory confirmation > 3 days after admission [24,25].

g. As reported by Jhung et al in publication [24].

h. Derived from Taylor et al’s Figs 1 and 2 [25].

i. As reported by Taylor et al, 8.9% of HAI cases overall had fatal outcome for which influenza was direct or contributing cause; same was assumed for the 2010–11 season [25].

j. Assuming 60% of all HAI outcomes may be attributed to HCWs (i.e. values in the preceding row multiplied by 0.6).

k. Derived as: $(patient\;HAI\;deaths\;attributed\;to\;any\;HCW)\;x\;\frac{\left(100\%-VC\right)}{\left(100\%-VC)+[VC\;x\;(1-\;VE)\right]}$; where VE = vaccine efficacy.

l. Indirect VE against any HAI outcome in patients assumed to be equivalent to the direct VE in HCWs assumed at 60% [21]. Thus, values in the preceding row multiplied by 0.6.

m. NNV derived as: $\;\frac{Number\;of\;unvaccinated\;HCWs}{patient\;HAI\;deaths\;preventable\;by\;HCW\;vaccination}$.

The Canadian network reported by Taylor et al consisted of 54 hospitals that conducted surveillance for hospital-acquired influenza across six seasons (2006–2012), with a variable number of participating hospitals each year [25]. The 2010–11 season had the highest number of hospital-acquired influenza cases and for consistency with the Jhung study, we focused analysis on that season. Together this Canadian hospital-based surveillance network comprises 35% of all hospital bed-days in Canada. There are ~500,000 hospital workers in total in Canada. During the 2010–11 season, 35 (65%) of the 54 hospitals in the network contributed to influenza surveillance activities (personal communication, G. Taylor and D Gravel) [25]. Assuming a 1:1 ratio of HCWs to hospital bed-days, we estimated the number of HCWs employed across the network during the 2010–11 season to be 35% X 65% of the 500,000 hospital workers in Canada (i.e. ~113,000). We further assumed that HCW influenza vaccine coverage across the network was 40%, as documented elsewhere for Canadian hospitals [38,39]. During the 2010–11 season, the study identified 257 healthcare associated influenza cases of which 32% (82) were acquired in hospital (versus long-term care). Applying the 8.9% influenza-associated case fatality reported overall by Taylor to the 2010–11 season, yields 7 hospital-acquired influenza deaths identified across the 35 participating hospitals that season (Table 3).

Of these 27 US and 7 Canadian deaths in 2010–11, we estimated that 8 and 4 respectively were attributed to unvaccinated HCWs and 5 and 2 respectively could be prevented through HCW vaccination, assuming HCW-attributable risk and vaccine efficacy each of 60% (Table 3).

Based on these data, the number of hospital staff that would need to be vaccinated (NNV) to prevent a single patient death was similar in the Canadian and US settings at 32,688 and 32,819, respectively (Table 3). The NNV would be higher with lower VE or lower HCW-attributable risk. If instead of 60%, VE in HCWs was assumed to be 40% or less as reported by several studies in Canada and the US for the 2010–11 season [40–42], the NNV would increase to at least 54,193 and 61,111, respectively. Even allowing that as much as 80% of hospital-acquired influenza deaths were due to HCWs, the NNV in hospitals would still range between 24,516 (Canadian network) and 24,614 (US network) for a VE of 60%, and between 40,645 and 45,833 for a VE of 40%. Applying the highest adjustment factor (5.2) for under-detection of influenza reported by Reed et al [43] for elderly patients admitted to the US hospital network in 2010–11, the NNV would still range between 6286 and 6311 for a VE of 60% and between 10,241 and 11,752 for a VE of 40%.

## Discussion

Policies of enforced HCW influenza vaccination are gaining popularity in North America, bolstered primarily by four cRCTs broadly interpreted to prove substantial patient benefit. Our analyses, however, show that these policies lack a solid empirical underpinning. Although RCTs represent the gold-standard study design for assessing interventions, unblinded cRCTs are at greater risk of bias and where their conclusions appear implausible caution is required in their interpretation, particularly if used to guide policies that abrogate individual rights. In that regard, each of the four cRCTs used to champion compulsory HCW influenza vaccination policies reports patient benefits that are mathematically impossible under any reasonable hypothesis of indirect vaccine effect. Whereas it is assumed on the basis of these studies that unvaccinated HCWs place their patients at great influenza peril, we show through detailed critique and numerical recalibration that these impressions are exaggerated.

*Eq 1* makes plain the principle of dilution—a relatively simple concept, whose central importance in assessing the reliability of the cRCT evidence seems to have been overlooked by policy analysts to date. We detail it here to expose an obvious incongruity: reported percentage reductions that are greater for less specific outcomes or that exceed increases in vaccine coverage cannot be accepted as valid demonstration of vaccine benefit. To reinforce this understanding, we underscore that other (non-cluster) RCTs that have explicitly reported the effects of dilution have shown its substantial impact. For example, VE against specific laboratory-confirmed influenza vs. non-specific ILI in RCTs involving HIV-negative pregnant mothers was 50% vs. 4% [44]; in HIV-positive adults was 75% vs. -7% [45]; in adults receiving live attenuated influenza vaccine was 42% vs. 4% [46]; and in children receiving quadrivalent vaccine was 59% vs. -3% [47]. In each of these real RCT examples, VE estimates for ILI were at least 10–12 times lower than for laboratory-confirmed influenza. These substantial differences in RCT-derived VE for laboratory-confirmed influenza vs. ILI are in the context of comparing wholly vaccinated to wholly unvaccinated participants and do not require the further downward adjustment associated with partial vaccine coverage as in the cRCTs to assess increased but incomplete HCW vaccine coverage between intervention and control sites. To maximize the predicted benefit to patients we assumed that 35% of ILI could be attributed to influenza, resulting in a VE against ILI that was just three times (not as much as 10–12 times) lower than for laboratory-confirmed influenza. In other words, in our optimistic projections we have substantially under-estimated dilution effects in favour of over-estimating potential patient benefits defined by non-specific outcomes. Even so, the cRCTs to assess indirect patient benefit through increased HCW vaccination have reported estimates that exceed by several-fold even our most optimistic projections of plausibility and are thoroughly inconsistent with the principle of dilution.

Although the derivations we present based on *Eq 1* may seem simplistic, they should be understood to have *a priori* over-estimated potential patient benefit; any other conditional probabilities (decimal multipliers) included as “chain of event” transmission parameters within a more complex model would serve only to further reduce the patient benefits we have optimistically projected as attributable to HCW vaccination. This is shown in the dynamical simulations of van den Dool et al [48,49] incorporating additional parameters beyond direct VE and vaccine coverage, such as the likelihoods of close or casual contacts between different potential sources (HCWs or patients) involved in successful transmission. In both the nursing home and hospital ward simulations by van den Dool et al [48,49], assuming a direct VE of 73% in HCWs, authors showed in their dynamical models that increasing HCW vaccine coverage from zero to 100% reduced the influenza attack rate in patients by about 40%, an indirect VE that is a little more than half the direct VE in HCWs [48,49]. In all of the acute care simulations that varied other parameters but not the direct VE, vaccinating all HCWs gave lower indirect protection to patients (30–50%) than direct protection (73%) to HCWs [48].

The simulations by van den Dool et al are based on small facility settings (e.g. 30-bed nursing home, 24-bed hospital ward) and result in average HCW infection rates (absent vaccination) of 50% in the hospital scenario—more than double the highest (serologically-defined) estimate measured empirically by Elder et al among unvaccinated hospital workers [50], and more than 50 times greater than the rate of PCR-confirmed symptomatic influenza identified among hospital workers during a three-year active surveillance initiative [51,52]. It is difficult to conceive of one in two HCWs becoming infected each year and by furthermore assuming uniform transmission risks whether from symptomatic or asymptomatic infection and that all infected HCWs continued to work and took no other precautions throughout their full infectious period, the van den Dool modeling likely also over-estimates the burden to patients [48,49]. Accordingly, the modeling findings by van den Dool et al should not be interpreted or extrapolated at face value but do nevertheless reinforce the important general message that incorporation of additional multiplicative probabilities reduces indirect VE compared to direct VE. With our assumption that indirect VE in patients is instead equivalent to direct VE in HCWs, our simple model will have therefore prominently over-estimated predicted patient benefits more so even because the direct VE of 60% [21] we have assumed is itself likely an over-estimate. Several studies [40–42,53–59] and a recently updated meta-analysis [60] report lower direct VE (<40%, on average), even in healthy young adults, against A(H3N2)—the subtype that is responsible for nearly 80% of influenza-related deaths, particularly in the elderly [61].

Despite assumptions designed to substantially over-estimate indirect patient benefit, the four cRCTs reported reductions in all-cause mortality among patients that were 6- to 15-fold greater than predicted (Table 1). Even allowing that as much as 50% of ILI and 30% of all-cause mortality across the season could be due to influenza, we found the reductions reported by Hayward et al [13] to be five times greater than predicted, beyond what could be realistically attributed to HCW vaccination. The lower limits of the 95% confidence intervals in pooled meta-analyses by the US CDC and Cochrane groups also exceeded predicted mortality reductions by 4- to 12-fold (Table 1) [15,16]. Even if confidence intervals around the various non-specific outcome estimates overlap, they cannot be cited independently of implausible point estimates. Confidence intervals are meant to reflect variation around true effect size based on the normal fluctuation arising with random sampling, assuming no other bias is influencing the measured effect [62]. However, the confidence interval is directly dependent upon the observed point estimate and the study sample size. To invoke the lower limit of the confidence interval to explain away otherwise implausible point estimates is to conflate the two separate possibilities of random variation (lack of precision, a statistical consideration) and systematic bias (lack of validity, an epidemiological consideration). When the point estimate is not valid (e.g. when there are concerning epidemiological signals of implausibility or bias), the confidence interval directly dependent upon it is also not valid. Random error may add to systematic error, but it provides no defense against an apparent lack of validity in a study’s main finding.

Cluster RCTs are at greater risk of bias to explain findings than trials with individual randomization. As highlighted by the Cochrane group, the cRCTs showing patient benefit through increased HCW vaccination in LTCFs suffered multiple methodological issues including failure to conceal group allocation (i.e. blinding), poor performance in increasing HCW vaccine coverage, insufficient power to assess specific influenza outcomes and the possible influence of selection bias [16]. None of the trials provided information on other co-interventions such as hand washing, quarantining, or requesting/requiring HCWs with ILI to stay home or mask. Education and other promotional efforts delivered as part of a program to increase voluntary vaccination within intervention sites may have simultaneously enhanced awareness of and compliance with these other more broadly protective practices, which may have then confounded or compounded the vaccine effects.

The effects of these non-vaccine factors may be particularly evident in the chosen analysis periods prior to influenza circulation in at least two of the four cRCTs. HCW influenza vaccination cannot prevent ILI or death in patients for which influenza is not somehow implicated and therefore cannot account for differences between intervention and control groups before the period of influenza virus circulation. It is then likely that the confounding factors or co-interventions driving those pre-existing differences between groups had persistent effects through the rest of the study period. This type of confounding in reporting indirect VE against non-specific patient outcomes is reminiscent of the bias previously identified by Jackson et al in reporting significant VE against all-cause pneumonia and mortality in seniors themselves, not only during the influenza period, but also before and after [63]. To further investigate and quantify the impact of this potential bias, future trials should include control outcomes that are clearly not due to influenza and are not preventable by vaccine, and should place greater emphasis on laboratory confirmation when assigning influenza attribution. In the meantime, it should be well understood that HCW vaccination could have only accounted for a small but unknown proportion of the total patient benefits reported by the four cRCTs; most of the observed reductions in non-specific patient outcomes must have instead been due to accompanying non-vaccine protective measures or otherwise reflect unrecognized biases.

Supporters of compulsory policies have cited other studies, including an additional cRCT conducted by Riphagen-Dalhuisen et al in acute care facilities [64]. The main objective of that trial, however, was to assess whether a multi-faceted intervention could increase HCW influenza vaccine coverage. Influenza illness in patients was only assessed as a secondary outcome, without standardized surveillance and based only upon retrospective assessment through computerized discharge notes. The authors reported a significant 50% reduction in the combined outcome of pneumonia and/or influenza among patients in the department of medicine despite just 8% difference in HCW vaccine coverage between the intervention and control groups. As per above, it is not possible to attribute this magnitude of reduction in patient outcome to such minimal change in HCW vaccine uptake, particularly since many pneumonias do not have influenza as a root cause and are not preventable by influenza vaccine, directly or indirectly. The US CDC review also considered four observational studies that have reported indirect patient benefit through HCW influenza vaccination [15]. These observational studies are even more susceptible to bias: each reports percentage reductions (three of four in relation to ILI) that substantially exceed increments in vaccine coverage and are irreconcilable with the principle of dilution [52,65–67].

It is in generalizing beyond the context of voluntary programs in LTCFs to compulsory vaccination of all acute care staff that incongruities in the cRCT evidence become most apparent. A literal extrapolation of the heretofore unchallenged NNV estimate of 8 to all hospital staff leads to the untenable conclusion that unvaccinated HCWs pose a risk to their patients that is tantamount to a 1918-style influenza pandemic every year (within the nosocomial setting alone). To recalibrate these utterly implausible impressions, we drew upon published hospital-based surveillance networks in the US and Canada, focusing on their real data for patient mortality as the outcome most clinically and ethically relevant to the argument for enforced HCW vaccination. This showed that more than 32,000 hospital staff would need to be vaccinated before a single patient death could potentially be averted—4000-fold greater than estimated by Hayward et al [13]. These hospital-based influenza surveillance networks may have missed some hospital-acquired cases but severe cases culminating in death were less likely to have been missed than milder, non-fatal cases or those that were admitted but community-acquired [24, 25]. Even if we nevertheless apply the highest adjustment factor (5.2) for under-detection of influenza as reported by Reed et al for elderly patients admitted to hospital [43] as if it applied to all hospital-acquired influenza, the NNV estimate would still exceed 6000, or about a thousand-fold greater than reported by Hayward et al [13]. We assumed that 60% of hospital-acquired influenza was attributable to HCWs. This may be considered an underestimate of their contribution to original and onward patient transmission but given the role of other influenza sources (i.e. patients and visitors) in the dynamic and porous hospital setting [26–28], our choice more likely represents an over-estimate. We nevertheless explored up to 80% HCW attribution in sensitivity analysis. If policies of enforced HCW vaccination are further expanded beyond hospitals, to also include ambulatory care staff, the NNV to prevent patient death is expected to be even greater, given shorter and less intense contact patterns and risk compared with the care given to bedridden patients. As per above, our assumption that direct VE in HCWs is equivalent to the indirect VE in patients will have prominently under-estimated the NNV. Our NNV estimates should thus be considered minimum values indicative of a more realistic order of magnitude compared to Hayward et al [13], rather than an exact measure of impact. The latter would require actual scientific quantification of the HCW-attributable risk and the fraction indirectly preventable by HCW vaccination, by healthcare setting. However, those values are absent in the published literature for any setting.

Other personal benefits to HCWs or their families, or to their institutions through possible reductions in worker absenteeism, were not factored into our analyses since these do not constitute the main ethical imperative for enforced vaccination of HCWs compared to other citizens or occupational groups. We did not consider the emerging evidence of negative effects from prior vaccination on subsequent influenza vaccine protection or pandemic risk, or the potential liability to institutions or organizations in the event of vaccine-associated harm under compulsory or coercive policies [59,68–77]. Our analyses did not consider the impact of fully implementing other infection control measures (e.g. staying at home when ill, masking when ill, hand hygiene) which, even acknowledging the uncertainty in the success of these interventions [78–81], would reduce the burden of patient deaths preventable through HCW vaccination (further increasing the NNV). We did not address the benefits to patients from more extreme measures such as season-long, asymptomatic mask wearing, offered as the only alternative to vaccine under some HCW policies. If the epidemiologic evidence for indirect patient benefit through HCW influenza vaccination is considered flawed the further hypothesis of indirect patient benefit through obligatory masking of unvaccinated but asymptomatic workers is completely conjectural. There are, of course, other logical fallacies in policies requiring the masking of unvaccinated HCWs throughout the influenza season to protect patients. Given the overall burden of nosocomial respiratory infections, including other serious non-influenza viruses such as RSV or human metapneumovirus, influenza vaccination should be considered insufficient. On average more than 40% of vaccinated HCWs remain vulnerable to seasonal influenza overall [21]—more than 60% in relation to the most dangerous A(H3N2) subtype [60]—and in that context, a coherent prevention policy to reduce risk to patients to the extent possible would dictate the wearing of masks by all HCWs, vaccinated or unvaccinated, for the duration of the winter respiratory season. We are unaware of such extreme policies anywhere to date.

Through this detailed critique and quantification of the evidence we conclude that policies of enforced influenza vaccination of HCWs to reduce patient risk lack a sound empirical basis [82]. In that context, an intuitive sense that there may be *some* evidence in support of *some* patient benefit is insufficient scientific basis to ethically override individual HCW rights. While HCWs have an ethical and professional duty not to place their patients at increased risk, so also have advocates for compulsory vaccination a duty to ensure that the evidence they cite is valid and reliable, particularly in the absence of good scientific estimates of patient impact. The diversion of resources from more evidence-based efforts and other important but less tangible costs related to loss of trust and credibility also need to be considered, including the implications for other immunization programs and workplace policies. Although current data are inadequate to support enforced HCW influenza vaccination, they do not refute approaches to support voluntary vaccination or other more broadly protective practices such as staying home or masking when acutely ill.

## Supporting Information

S1 Appendix Practical grocery cart example of the principle of dilution and the relationship between outcome specificity, percentage reduction and coverage. (PDF)
