# Supplementary material for: Influenza Vaccination of Healthcare Workers: Critical Analysis of the Evidence for Patient Benefit Underpinning Policies of Enforcement
Source: PLoS One. 2017 Jan 27;12(1):e0163586. doi: 10.1371/journal.pone.0163586 (PMC5271324; doi:10.1371/journal.pone.0163586)
Supplement: S1 Appendix — (PDF) [file pone.0163586.s001.pdf]

S1 Appendix.
Practical discount coupon example of the principle of dilution and the relationship between outcome specificity, percentage reduction and coverage

Most shoppers intuitively know that the percentage reduction from a discount coupon will always be greatest in relation to the featured (specific) product, and lower when calculated in relation to the entire all-item shopping bill. A "60%-off" coupon for a featured item cannot give "60%-off " the total grocery bill inclusive of non-featured items. This practical grocery cart example, while intuitive and commonplace, follows the basic equation for the principle of dilution, incorporating the relevant compound probabilities shown below:

$$\%\,\boldsymbol{reduction\ on\ shopping\ bill} \\ = \%\,reduction\ from\ coupon\ x\ \%\ targeted\ item\ in\ bill\ x\ \%\ coverage\ of\ the\ coupon$$

Figure illustrating the effects of a discount coupon for "60%-off" a featured chicken item:

**Grocery Cart Check-Out: No Coupon**		
**Meat Items**		**$90**
Chicken X 3	$30	
Steak	$40	
Pork	$20	
Eggs & Dairy		$30
Fruits & Vegetables		$70
Bread & Cereals		$25
Other items		$85
		TOTAL: $300

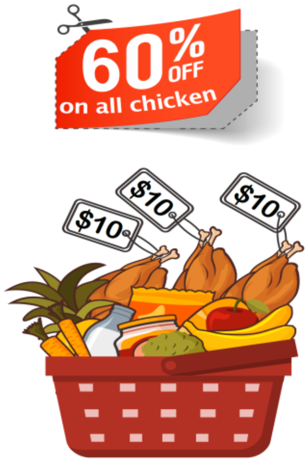

**Grocery Cart Check-Out: + "60%-off on all chicken" Coupon**		
**Meat Items**		**$72**
**$30 – (60% X $30) =**		
Chicken X 3	$12	
Steak	$40	
Pork	$20	
Eggs & Dairy		$30
Fruits & Vegetables		$70
Bread & Cereals		$25
Other items		$85
		TOTAL: $282

"60%-off" coupon valid on all chicken purchases:
60% reduction on bill for chicken items ($18/$30)
20% reduction on bill for meat items ($18/$90)
6% reduction on bill for all grocery items ($18/$300)

<u>Grocery cart example: discount coupon for "60%-off" a featured chicken item:</u>

In the above grocery cart example consisting of three $10 chicken items (total value $30), sundry other non-chicken meat (valued at $60), and non-meat items (valued at $210) the all-item grocery bill without the discount coupon is $300.

The chicken items targeted by the "60%-off" coupon represent one third ($30/$90=33%) of overall meat items purchased and a smaller proportion ($30/$300=10%) of the all-item (meat and non-meat) grocery bill. As additional items against which the chicken coupon has no effect are added to the grocery cart (i.e. as chicken comprises a smaller proportion of the all-item grocery bill), the chicken coupon provides a lower percentage reduction on the *total* shopping bill.

In accordance with the principle of dilution, the coupon that is effective for 60% reduction off the cost of all three $10 chicken items (i.e. an $18 saving on the total $30 chicken cost) confers a lower

20% reduction in relation to the cost of overall meat items (i.e. $18/$90) and even lower 6% reduction in relation to the all-item grocery bill (i.e. $18/$300).

Percentage reduction also varies with coupon coverage. If the "60%-off" discount coupon were only "good for" a single $10 chicken purchase rather than all three chicken items in the cart, then percentage reductions would be adjusted accordingly in relation to the reduced coupon coverage (by one-third). There would instead be 20% reduction in relation to the total chicken cost (i.e. $6/$30), 7% reduction in relation to overall meat cost (i.e. $6/$90) and 2% reduction in relation to the all-item grocery bill (i.e. $6/$300).

Indication of error based on the principle of dilution

In measuring coupon effects, the percentage reduction must always be **proportional to the dilution of the featured item by non-featured items in the grocery cart and to the coverage of featured items by the coupon** (e.g. all chicken items or limited to only one or two or some other proportion of all chicken items purchased). Where this is not observed, there is a *de facto* error.

If at the check-out counter, the cashier takes the "60%-off chicken" coupon that the customer provides, and charges the customer only $120 on a full grocery cart bill originally valued at $300 (a 60% reduction in the all-item grocery cost rather than "60%-off" chicken items) then the customer knows there has been an error.

The customer may be very pleased to accept this error at face value but no matter how many times the cashier or others may insist that this is due to the rebate given by the coupon, the customer would know that it is impossible to legitimately attribute to the coupon. A "60%-off" coupon specific for chicken cannot possibly give "60%-off" the cost of a total grocery bill inclusive of other non-chicken items, and the more that other items contribute to the total grocery bill, the less possible this can be. The effects of the discount coupon specific for a featured product must always decrease as more non-featured items contribute to the grocery bill.

Relevance to influenza vaccine benefit

The same principle of dilution applies in attributing percentage reductions in non-specific outcomes to influenza vaccination. Influenza vaccine is only effective against influenza virus and provides no benefit against other causes of illness or death unrelated to influenza virus. As more non-influenza causes contribute to the outcome considered, the lower must be the percentage reduction attributed to vaccine effects. Where that is not observed, there is a *de facto* error in claiming vaccine benefits.

In the above example, instead of "chicken" substitute the word "influenza"; instead of "coupon" substitute the word "vaccine"; instead of "overall meat items" substitute the word influenza-like illness (ILI); and instead of "all-item grocery bill" substitute "all-cause mortality" and the same pattern would have to be true.

The percentage reduction from the rebate (coupon or vaccine) must always be higher in relation to the outcome that is specifically targeted (chicken or influenza) and lower as more non-targeted (non-specific) conditions (all-item groceries or all-cause mortality) are included in summarizing benefit.

In our manuscript, the plausibility of percentage reductions reported in non-specific patient outcomes (i.e. ILI and all-cause mortality) attributed to differences in healthcare worker influenza vaccine coverage are examined in detail based on this universal and basic mathematical principle of dilution.
